# Approximate image synthesis in optical coherence tomography

**DOI:** 10.1364/BOE.420992

**Published:** 2021-05-12

**Authors:** Callum M. Macdonald, Peter R. T. Munro

**Affiliations:** Department of Medical Physics and Biomedical Engineering, University College London, Gower Street, London WC1E 6BT, UK

## Abstract

Full-wave models of OCT image formation, which are based on Maxwell’s equations, are highly realistic. However, such models incur a high computational cost, particularly when modelling sample volumes consistent with those encountered in practice. Here, we present an approximate means of synthesizing volumetric image formation to reduce this computational burden. Instead of performing a full-wave scattered light calculation for each A-scan, we perform a full-wave scattered light calculation for a normally incident plane wave only. We use the angular spectrum field representation to implement beam focussing and scanning, making use of an assumption similar to the tilt optical memory effect, to approximately synthesize volumetric data sets. Our approach leads to an order of magnitude reduction in the computation time required to simulate typical B-scans. We evaluate this method by comparing rigorously and approximately evaluated point spread functions and images of highly scattering structured samples for a typical OCT system. Our approach also reveals new insights into image formation in OCT.

## 1. Introduction

Optical Coherence Tomography (OCT) is a well established optical technique which enables three-dimensional imaging of scattering media, and has enabled numerous applications in biomedicine and beyond [1–4]. Further development of this technology towards greater quantitative imaging capabilities will likely require a computational imaging approach, using measured OCT data in tandem with optical wave propagation models. Realistic models are thus crucial to the development of such quantitative techniques and must achieve a suitable trade-off between accuracy and computational efficiency.

When modelling image formation in spectral-domain OCT, we require a means of computing the interaction of a broadband source-field with an arbitrary sample and, in particular, the resulting scattered field. A number of different modelling approaches have been employed in OCT [5–19], which invoke a variety of approximations in accordance with their application. Broadly speaking it is possible to separate existing, non full-wave, models into two categories: those based on a point spread function (PSF) or equivalent approach, and those based on the Monte Carlo method. Models based on a PSF formalism can draw on considerable literature on image formation in coherent optical microscopes (e.g. [20]). When applied to turbid media, approximations are made about how light interacts with a complex sample. This often involves the first-order Born approximation, which assumes that light undergoes a single scattering interaction on its journey between source and detector, allowing images of complex samples to be built up assuming linear superposition [21]. This has been applied in inverse scattering approaches which use OCT data [10], or when making use of linear filter theory [18] and coherent transfer functions [19]. In some applications it is important to include the effect of sample-induced attenuation and multiple scattering in the simulated image, which can degrade the PSF [22]. This can be approximately treated by including a depth dependent attenuation factor [23], or by using the extended Huygens-Fresnel formalism [8,24]. Here, attenuation and multiple scattering are represented using scatterer ensemble averaged values and are not valid for deterministic scatterer distributions.

The Monte Carlo method of modelling light propagation in biological tissue [14] has been used as the basis for numerous OCT image formation models (for example [7,11,12,25–27]). Monte Carlo modelling is the gold standard in various applications in biomedical optics and has revealed a range of important insights in OCT image formation. However, Monte Carlo based models also possess limitations. For example, Monte Carlo models represent tissue on the basis of spatially averaged values of scattering coefficient and anisotropy, which are assumed to “extend uniformly over small units of tissue volume” [14]. Also, although the technique is continually being improved along these lines, Monte Carlo methods do not naturally facilitate explicit modelling of wave phenomena including coherence and interference.

In order to further advance applications which rely on an understanding of image formation in OCT, we require increasingly realistic representations of the underlying physics. “Full-wave” models, that is, models which directly satisfy Maxwell’s Equations, offer a means to overcome the limitations inherent to PSF and Monte Carlo based approaches. Full-wave analysis of the field within the sample has been performed in [28–32], although these approaches do not include an image formation model. Full-wave models which do include rigorous models of the illumination and detection optics of the OCT system, as well as light propagation in the sample, have been introduced in two- [17,33] and three-dimensions [16]. These models are applicable to general sample structures and require the use of rigorous numerical Maxwell’s equations solvers, for example, employing Finite Difference Time Domain (FDTD), the Pseudo-Spectral Time Domain (PSTD) and Born series [34] methods. Such methods are implicitly capable of treating wave phenomena such as multiple scattering, sample induced aberration, and polarisation. Yet, due to their substantial computational requirements, these models have only recently become feasible for large samples (*i.e.*, samples of a meaningful size in a biomedical context). The inherent computational burden associated with the PSTD and FDTD techniques arises from the fact that updates of the electric and magnetic fields must be computed at all locations within a medium at every discrete time step, and with sufficient granularity in both time and space to satisfy fundamental criteria [35]. In this study we seek to exploit certain wave phenomena and reduce this computational burden, whilst still retaining the benefits of these full-wave models.

**Fig. 1.**
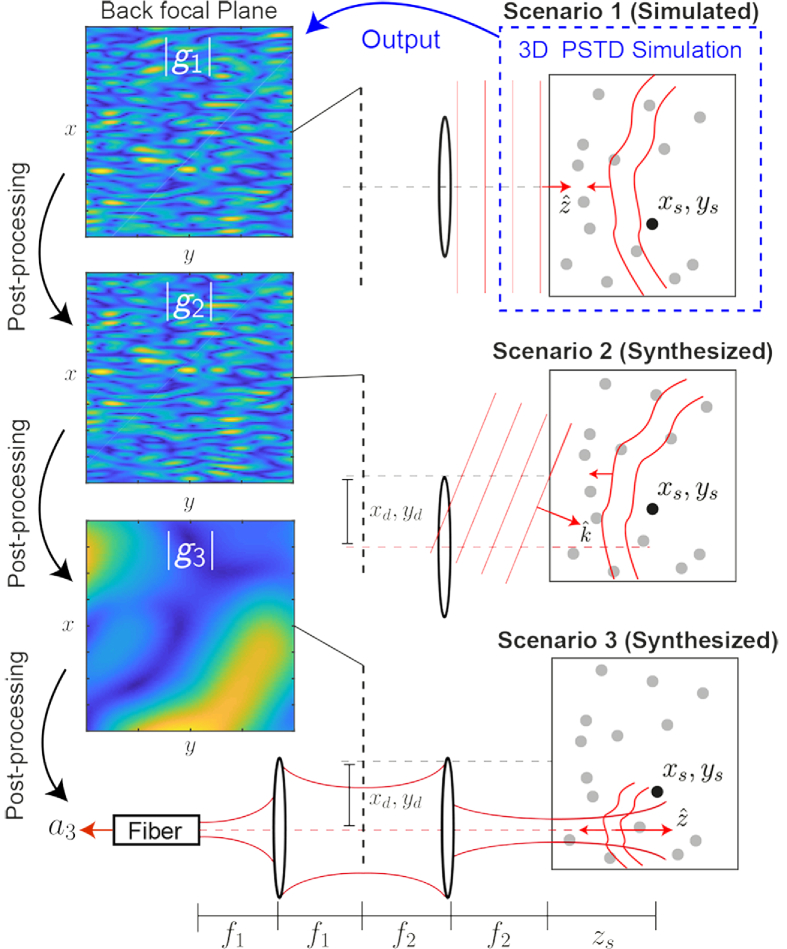
Details of three scenarios under consideration, and examples of the corresponding scattered fields in the back focal plane of the objective lens. Scenario 1 - Normally incident plane wave illumination of the sample. Scenario 2 - Tilted plane wave illumination. Scenario 3 - Focussed illumination of the sample and detection with a single-mode optical fiber. Scenario 1 is simulated by a 3D PSTD wave propagation simulation. Scenarios 2 and 3 are then synthesized by post-processing the field $g_{1}$.

In this work we utilize a PSTD solver previously described in Ref. [16] for computing the field scattered by an object. Objects are described by their spatial refractive index distribution, and thus may be very general. OCT image formation is computed by calculating how the field scattered by these objects interferes with light from a reference arm after being coupled into an optical fiber. The typical operation of this OCT forward model is somewhat burdensome when imaging a large sample, as multiple independent simulations are required, in particular, one for each scan-position of the illuminating beam (i.e., for each A-scan) [16]. Fortunately, for a given scan position of the focussed beam, we can omit regions of the sample not illuminated by the beam from the computation. While this reduction in effective sample volume substantially reduces A-scan computation time, there still remains significant overlap in the sub-volumes which make up adjacent A-scans, leading to large effective sample volumes being simulated when the sum of all simulated sample volumes is calculated.

In this study, we seek to exploit the redundancy that exists between the computation of light propagation in overlapping scan positions by employing a different approach to modelling the scanning process. In the approach presented here, which we refer to as “synthetic scanning”, we perform only a single PSTD simulation of the entire sample using normally-incident plane wave illumination, instead of one simulation for each scan position of a focussed beam. The scattered field computed for the incident plane wave is then used to synthesize the scattered field that would have been observed using focussed illumination at each scan position. These synthesized fields are then used to compute the field coupled into the sample arm of the OCT interferometer, for each synthesized scan position of a C-scan. Our method reduces the required computation time of the PSTD model, relative to the direct method of simulation, by an order of magnitude when simulating OCT B-scans. This new approach also has the advantage of being able to recompute the OCT signal for arbitrary illumination beam profiles without needing to re-run the PSTD algorithm, which is the most computationally intensive part of the image formation model. In the following sections we outline the algorithm which synthesizes scanning-mode acquisition. We then numerically compare the accuracy of this method to the more computationally-intensive, yet more rigorous, direct simulation approach.

## 2. Synthesizing scanning-mode signals

In this section we derive an approximate relationship between the scattered field resulting from normally incident plane wave illumination, and the scattered field resulting from a focussed illumination beam. This relationship is derived by first considering the analytic solutions for both scenarios when imaging a single point scatterer. However, the derived relationship will later be applied to synthesize the focussed illumination scenario from the plane wave scenario, using the output of a full-wave 3D PSTD simulation. We begin by describing the problem for a monochromatic source. When modelling image formation in Fourier-domain OCT, the below steps will need to be repeated for each wavenumber, k, in the spectrum of the simulated broadband source. We assume a low Numerical Aperture (NA) objective and that depolarization due to focussing is negligible, allowing for a scalar description to suffice in this scenario.

Consider first Fig. 1 where we display the geometry of the problem. In scenario 1, a monochromatic plane wave travelling in the +z direction, with phase exp[ikz], illuminates the sample (note that we use the −iωt time convention). An objective lens of focal length $f_{2}$ collimates the scattered field onto its back focal plane, resulting in a field which we denote $g_{1}(x,y,k)$, where x,y are co-ordinates in the back focal plane. In order to build an approximate relationship between scenario 1 and scenario 2, we first assume the sample medium contains only a point scatterer at location ($x_{s},y_{s},z_{s}$), where $z_{s}$ is measured relative to the focus in the sample region. Propagating the scattered field from the plane of the point scatterer onto the back focal plane (assuming free-space propagation), we then have:

(1) $$g_{1}(x,y,k)=\frac{1}{i\lambda f_{2}}\exp\left[i2kz_{s}\right]\text{exp}\left[\frac{-ikz_{s}}{2f_{2}^{2}}(x^{2}+y^{2})\right]\text{exp}\left[\frac{ik}{f_{2}}(x_{s}x+y_{s}y)\right]\;.$$

This expression is derived from Eq. (6) in Ref. [36], neglecting the finite size of the lens, and is valid for scatterers located within the typical designed depth of focus in an OCT system. In Fig. 1 scenario 2, we consider a plane wave illuminating the sample from a different (tilted) angle, where the wave vector now has transverse directional components $k_{x},k_{y}$, and where a laterally displaced lens (centered on $x_{d},y_{d}$) then collimates the scattered field onto the back focal plane, resulting in a new field $g_{2}$. Note that we will use the same coordinates x,y, to those in scenario 1, and these do not shift with the lens. Here we assume that, in terms of the incident field arriving at the point scatterer, the only change compared to scenario 1 is a phase shift. This phase shift can be described by the expected phase ramp of the tilted plane wave within the plane $z=z_{s}$. Regarding the magnitude of the field arriving at the point scatterer, this is assumed to be unaltered relative to the normally incident plane wave case. After some manipulation, the scattered fields in scenarios 1 and 2 for the same point scatter at location $\left(x_{s},y_{s},z_{s}\right)$ can be shown to be related by:

(2) $$\begin{array}{cc} g_{2}(x,y,k;x_{d},y_{d};k_{x},k_{y}) & =g_{1}\left(\bar{x}+\frac{k_{x}f_{2}}{k},\bar{y}+\frac{k_{y}f_{2}}{k},\;k\right)\;\text{exp}\left[\frac{iz_{s}}{f_{2}}(k_{x}\bar{x}+k_{y}\bar{y})\right]\\ & \times \;\text{exp}\left[-\frac{ik}{f_{2}}(x_{d}\bar{x}+y_{d}\bar{y})\right]\;\text{exp}\left[-ik_{x}x_{d}-ik_{y}y_{d}\right]\;, \end{array}$$

where we have used the substitutions $\bar{x}=x-x_{d}$, and $\bar{y}=y-y_{d}$. It can be seen from Eq. (2) that the field in the tilted plane wave case, $g_{2}$, can in fact be found by performing a translation operation on the field recorded in the first case, $g_{1}$, and in addition, applying a number of phase adjustment terms. Some or all of these phase terms disappear if the scatterer is in focus ($z_{s}=0$), or if the scan position is zero ($x_{d}=y_{d}=0$). Importantly, these phase factors in Eq. (2) have no dependence on the lateral position of the scatterer, ($x_{s},y_{s}$). This means that the field, $g_{2}$, can be synthesized without prior knowledge of the lateral location of the point scatterer, assuming we have a record of $g_{1}$. We note that the depth of the scatterer $z_{s}$ does appear in Eq. (2), however this term is slowly varying. The synthesized field, $g_{2}$, will thus be accurate so long as the scatterer is in the vicinity of the particular assumed value of $z_{s}$.

While Eqs. (1) and (2) were derived by considering a single point scatterer within the sample, our intention is to apply this relationship between the fields $g_{1}$ and $g_{2}$ to more general scattering samples. In particular, we intend to apply this relationship to synthesize $g_{2}$ using the field $g_{1}$ produced by a full-wave PSDT model of scattering in arbitrary samples. To do this with confidence, we must carefully consider the validity of each assumption made when deriving Eq. (2). First, in terms of the incident field arriving at a particular region of interest within a sample, we assumed that a well quantified phase change is experienced when tilting the incident plane wave, described by the expected phase ramp in any given plane with z= constant. In highly scattering samples the incident planar wavefront may become significantly perturbed. This alone does not prohibit the method from being applied to highly scattering samples (as it would if we were relying on a Born-like approximation). Rather, we only require the phase *change* at the region of interest to be correlated with the expected phase ramp when tilting the angle of an incident plane wave. This phase ramp may be expected to survive a significant degree of scattering, where in extreme cases the survival of such correlations has been characterized as an “optical memory effect” [37]. It is thus plausible that the assumed phase change may still be acceptable in highly scattering samples. It was also assumed above that the magnitude of the incident field arriving at the point scatterer does not change between the normally incident and tilted plane wave cases. Thus, this implicitly accounts for attenuation of the incident field whilst heading toward our layer of interest. Again, this is contrary to a Born-like approximation, which would assume free space propagation between the source and a region of interest within the sample. Next, in terms of the scattered field returning towards the detector from the point scatterer under consideration, it was assumed that the only difference between scenario 1 and scenario 2 is a phase change uniformly applied across the entire scattered wavefront. This assumption also implicitly allows for the scattered field to be perturbed through shallower regions of the sample when propagating towards the detector. In other words, considering our scatterer as a single point source, the (possibly perturbed) scattered field at any location will be altered in tandem with the change experienced by the point source. Finally, since the assumed phase change is a slowly varying function of the depth of the scatterer(s), $z_{s}$, the depth gating property of OCT is expected to further aid the synthesis step expressed in Eq. (2), by filtering out the contributions from other layers within the sample. Repeating the synthesis step with different values of the assumed depth of scatterers, $z_{s}$, will eventually allow a synthesized OCT image to be collated, these steps are described in more detail below. The validity of these underlying assumptions will be investigated further in the results section, however, we have outlined the reasons why the relationship expressed in Eq. (2) may remain viable even in highly scattering samples, and where the initial field $g_{1}$ is produced by a full-wave propagation model.

We now continue by considering the final scenario shown in Fig. 1 (scenario 3), where a focussed beam illuminates the sample at a laterally displaced scan position. Here we can consider the incident beam to be a superposition of a spectrum of plane waves having all possible incident directions within the NA of the objective lens. We describe this angular spectrum by ${\tilde{\phi }}_{f}$, where ${\tilde{\phi }}_{f}$ is the spatial Fourier transform of the complex source field in the sample space. Using the result from Eq. (2) above for each of these plane wave components, the resulting scattered field reaching the back focal plane in the focussed illumination case can be synthesized as

(3) $$g_{3}(x,y,k;x_{d},y_{d})=\int_{-k\text{NA}}^{k\text{NA}}g_{2}(x,y,k;x_{d},y_{d};k_{x},k_{y})\;{\tilde{\phi }}_{f}(k_{x},k_{y},k)\;dk_{x}dk_{y}\;.$$

Now that we can synthesize the field arriving at the back focal plane of the lens in the focussed-illumination case, we can also compute how this field would be coupled into an optical fiber, thus describing the sample arm signal of the OCT interferometer system. The complex coupling amplitude of a single-mode fiber, $a_{3}$, can be found by taking the inner product of the fiber mode projected into back focal plane, $\phi_{f}$, with the synthesized scattered field in the back focal plane, $g_{3}$. Remembering that the collection fiber mode is also translating in tandem with the illumination mode (as these are usually from the same optical fiber), we can write:

(4) $$a_{3}(k;\;x_{d},y_{d})=\int_{x^{2}+y^{2}<R_{a}^{2}}\phi_{f}(x-x_{d},y-y_{d},k)\;g_{3}(x,y,k;x_{d},y_{d})\;dxdy,$$

where $R_{a}$ is the aperture radius. We have thus demonstrated that if we first compute the scattered field arriving in the back focal plane resulting from normally-incident plane wave illumination, $g_{1}(x,y,k)$, we can synthesize the complex coupling amplitude of the scattered field coupled into the fiber in the focussed scanning case for each scan position of interest, $a_{3}(k;\;x_{d},y_{d})$. This set of steps is repeated for each of the wavenumbers we wish to sample as part of the OCT image formation simulation, and for each scan position $x_{d}$, $y_{d}$. The interference between fields from the sample, $a_{3}(k;\;x_{d},y_{d})$, and a reference arm signal, $a_{\text{mirr}}(k)$, can then be calculated and Fourier transformed, with respect to wavenumber, to produce an OCT A-scan for each scan position

(5) $$OCT(x_{d},y_{d},z)=F\left\{2R\left\{a_{3}(k;\;x_{d},y_{d})\;a_{\text{mirr}}^{*}(k)\right\}\right\}\;,$$

where F represents the Fourier transform operator, R denotes the real part, and a star denotes the complex conjugate. The benefit of this approach is that we are only required to run the PSTD simulation once, using plane wave illumination, as opposed to the more rigorous approach which would require a separate PSTD simulation for each scan position, with the interaction of the sample with a focussed illumination beam being explicitly modelled.

## 3. Results

### 3.1 Point spread function simulation

**Fig. 2.**
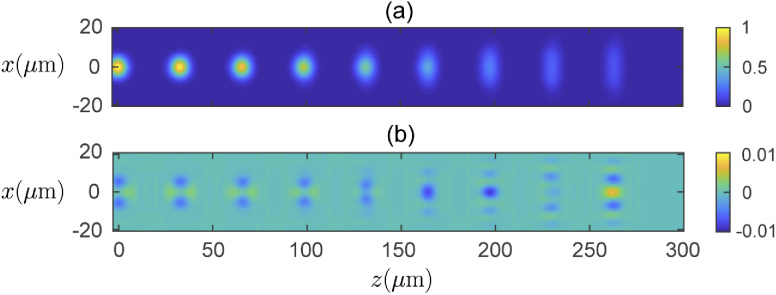
(a) - Synthesized PSF computed from single 3D PSTD simulation with normally incident plane wave illumination, normalized to the maximum value of the PSF (found at the scatterer in focus at z=0μm). (b) - Difference between reference and synthesized PSF magnitudes. Each scattering particle in the 3D simulation is separated by 32.93μm. All images are presented on a linear scale.

**Fig. 3.**
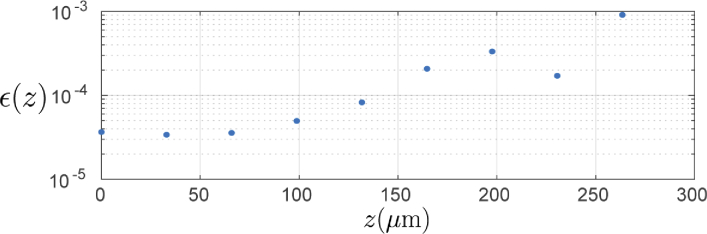
Error metric (Eq. (6)) plotted at the location of each point scatterer in the PSF sample along the axial direction.

We begin with a demonstration of the introduced method towards synthesizing the point spread function (PSF) of a typical commercial scanning OCT system. Here we model a Thorlabs, Inc. Telesto-II spectral domain OCT system with an LSM03 objective (focal length $f_{2}=36$mm, aperture radius $R_{a}=3.5$mm), and central wavelength $\lambda_{0}=1.3\mu$m. (see [38] for more details). The 3D sample consists of 9 point-like scatterers equally spaced along the optical axis, with a separation of 152 cells between each scatterer in the z-direction, where each cell in the simulation has a width of $\lambda_{0}/6$. A single 3D PSTD simulation was run for a plane wave normally incident on this sample. In the PSTD simulation, the plane wave is introduced to the medium as a temporal pulse, and in this particular sample the simulation iterates through 12070 discrete time steps to fully propagate the pulsed wavefront through the sample, including its interaction with the refractive index inhomogeneities (scatterers), and the resulting scattered field. From the recorded temporal scattered field, a time-independent field (equivalent to the continuous-wave case) is computed, and from the output of the PSTD algorithm the desired field $g_{1}$ is attained for 1024 discrete wavenumbers k making up the source spectrum [16]. In the post-processing step, for each wavenumber k, Eq. (2) is used to synthesize the field $g_{1}\to g_{2}$ for 63 discrete values of both $k_{x}$ and $k_{y}$, which are then integrated in Eqs. (3) and (4) to synthesize the complex coupling coefficient for the single mode fiber, $a_{3}$, and the corresponding OCT A-scan for a particular scan position ($x_{d},y_{d}$) using Eq. (5). Repeating these steps for each desired scan position produces an OCT B-scan, in this case there are 47 scan positions in the x-direction. Finally, these post-processing steps were repeated for a range of assumed scatterer depths of $z_{s}=0,50,100,150,200,250\mu$m, in order to accurately synthesize each depth region. A region of each of these scans was then used to populate the final B-scan. For example, the B-scan computed for $z_{s}=0$ is used to populate the final B-scan in the region −25μm≤z<25μm. The B-scan computed for $z_{s}=50\mu$m is used to populate the final B-scan in the region 25μm≤z<75μm, and so on. In Fig. 2 we show the resulting synthesized OCT image (Fig. 2(a)). This synthesized image is compared to a reference image computed using a more rigorous approach which explicitly models a scanned and focussed beam, and the discrepancy between the reference and synthesized images is shown in Fig. 2(b). The reference B-scan required a separate 3D PSTD simulation of a focussed beam propagating in the sample for each of the 47 scan positions, and thus required significantly more computational resources. A detailed comparison of computational requirements for each approach is left for the discussion section. The two PSF’s demonstrate close agreement, with the difference between the magnitudes of the two images shown in Fig. 2(b) being less than 1% of the maximum PSF magnitude for all locations. The accuracy of the field synthesis technique may be analyzed objectively using an error metric defined as:

(6) $$\epsilon (z)=\frac{\sum_{i}{\left|\left|OCT_{\text{ref}}(x_{i},z)\right|-\left|OCT_{\text{synth}}(x_{i},z)\right|\right|}^{2}}{\sum_{i}{\left|OCT_{\text{ref}}(x_{i},z)\right|}^{2}}$$

which sums over the lateral dimension for each value of the image depth, z. In Fig. 3 we show the error metric evaluated at each of the point scatterer z-locations where it is seen to remain below $10^{-3}$ for all points evaluated (deepest scatterer is at a location of z=263μm). It appears the error, although small, increases gradually as a function of depth.

### 3.2 Highly scattering sample

Next, we consider a more complex sample with a scattering structure of lateral width of 215μm and a depth of 250μm. This object is constructed from many closely packed scatterers which are designed to replicate the scattering properties of titanium dioxide spheres of 1μm diameter, and refractive index of n=2.488 at $\lambda_{0}$ (background refractive index is $n_{b}=1.42$), and have been arranged in the shape of the letter “B” oriented in the x-z plane. These scatterers are also distributed over a width of 40μm in the y-direction. The scattering coefficient in the regions containing the scattering particles is $\mu_{s}=11.4$${\mathrm{mm}}^{-1}$. More information on this numerical phantom may be obtained from [38]. See Fig. 10(a) for an image of the refractive index distribution of the scattering structure, which shows a projection of all of the scatters in the volume onto a single plane (x-z plane) for illustrative purposes only.

**Fig. 4.**
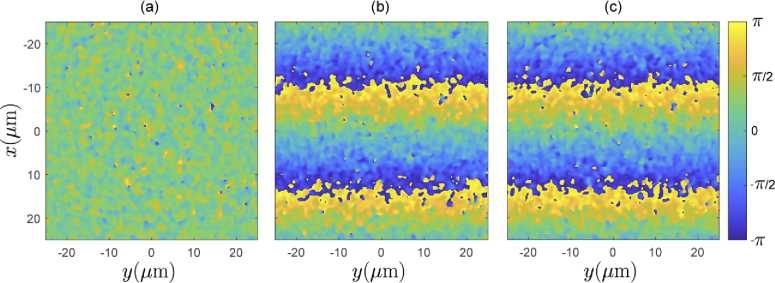
(a) Phase of the field arriving at the z=61.75μm plane within the sample for a normally incident wave. (b) Directly evaluated phase of the field for a tilted incident wave, with tilt angle $\theta_{i}=0.0457$ radians in the x-direction. (c) Phase of a synthesized field modified from (a) using the expected phase ramp induced by a tilted plane wave. All images shown for a wavelength $\lambda_{0}=1.3\mu$m.

**Fig. 5.**
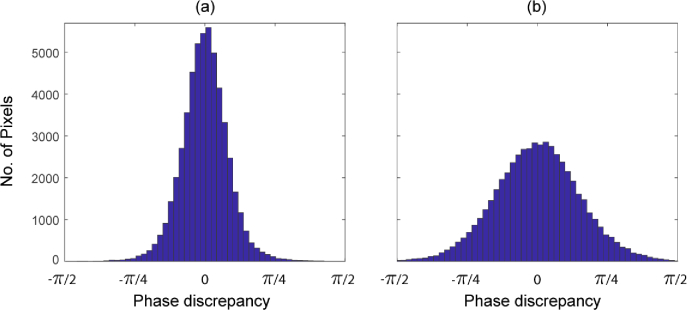
Histograms of the phase discrepancy (between the directly evaluated field and the synthesized field) within the 50×50μm region of interest at the z=61.75μm plane. (a) - Tilted pseudo-plane wave with $\theta_{i}=0.0076$ radians (b) - Tilted pseudo-plane wave with $\theta_{i}=0.0762$ radians.

**Fig. 6.**
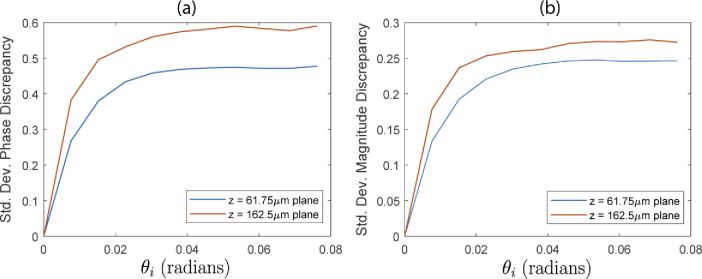
(a) - Standard deviation (radians) of the phase discrepancy (between the directly evaluated field and the synthesized field) over the 50×50μm region of interest at the z=61.75μm plane, $STD[\text{arg}(E_{\text{ref}})-\text{arg}(E_{\text{synth}})]$. (b) - Standard deviation of the field magnitude discrepancy over the same region of interest, $STD[||E_{\text{ref}}|-|E_{\text{synth}}||/<|E_{ref}|>]$.

To investigate the validity of our field synthesis technique for such scattering samples, we first explore the phase change within the sample due to a change in tilt angle of an incident plane wave. Recall that the field synthesis technique (described in Section 2) makes the assumption that this phase change is identical to that experienced by a the plane wave in the absence of scatterers. Figure 4(a) shows the phase of the field arriving at the plane z=61.75μm, within the sample, for a normally incident plane wave. The field was computed using the previously mentioned PSTD solver. We note that for technical reasons related solely to the PSTD method, in these examples we have simulated a weakly focussed incident beam, rather than a pure plane wave. The weakly focussed beam has negligible wavefront curvature over the region of interest in which we inspect the field, which corresponds to a 50×50 μm region in the center of the x-y plane. Figure 4(a) shows that the incident weakly focussed beam has been significantly perturbed due to propagation through the sample, resulting in a phase structure consistent with that of a speckle pattern. Figure 4(b) shows the phase at the same plane within the sample, but with the same weakly focussed beam tilted by an angle of $\theta_{i}=0.0457$ radians about the y-axis. A phase ramp is visually identifiable despite the speckle-like phase structure. Figure 4(c) shows the phase of the synthesized field. This is calculated by applying the phase ramp predicted by the tilted plane wave to the field from Fig. 4(a). The two phase images in Fig. 4(b) and (c) are visually consistent with each other despite having different speckle induced structure. To further analyze the agreement between the directly evaluated field (b), and the synthesized field (c) arriving at this layer within the sample, we plot histograms of phase discrepancy over the region of interest for two differing tilt angles. In particular, Fig. 5 shows histograms of the phase discrepancy for incident angles of $\theta_{i}=0.0076$ radians, and $\theta_{i}=0.0762$ radians. We see that for the lesser tilt angle, the phase discrepancy has a standard deviation of 0.2677 radians, whereas for the greater tilt angle, the standard deviation of the phase discrepancy increases to 0.4774 radians (≈π/6). Importantly, in both cases the mean of the phase discrepancy is zero. Figure 6(a) and (b) show the standard deviation of the phase discrepancy, and the magnitude discrepancy, respectively, for a range of incident tilt angles of the source. This has been evaluated over the same region of interest in the z=61.75μm plane, as well as a deeper layer of the sample in the z=162.6μm plane. The discrepancies are observed to increase both as function of incident angle of the weakly focussed source, and as a function of depth within the sample.

**Fig. 7.**
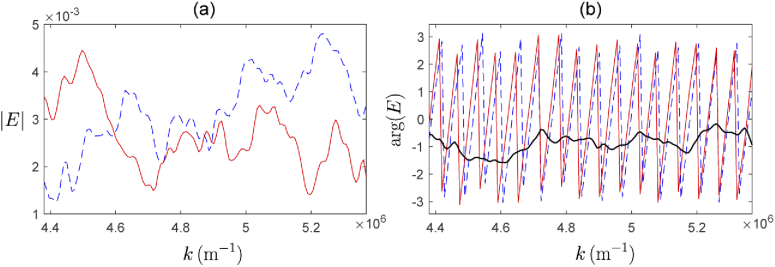
Example of directly evaluated field (solid red) compared to the synthesized field (dotted blue). Magnitude of field (a), and phase of field in radians (b), are plotted for the point x=0, y=0, z=61.75μm, as a function of wavenumber of the illuminating weakly focussed beam for an illumination angle of $\theta_{i}=0.0762$ radians. Also shown in (b) is the phase difference between directly evaluated and synthesized fields (solid black).

The preceding analyses are for monochromatic plane waves. Next we consider how the directly evaluated and synthesized fields within the sample vary as a function of wavelength. Figure 7 shows the magnitude and phase of the directly evaluated and synthesized fields at the point x=y=0 in the z=61.75μm plane, for a plane wave incident at an angle of $\theta_{i}=0.0762$ radians. In Fig. 7(a) we observe that the magnitude of the synthesized field may vary significantly at a given location, relative to the directly evaluated field. We also observe in Fig. 7(b) that the phase difference between the directly evaluated and synthesized fields may deviate by as much as π/2 in this case (solid black line), however, the overall rate of change of the phase as a function of wavenumber is closely related (number of cycles of the red and blue lines). This is important as it is this rate of change of the phase in the arriving fields (and thus also the scattered fields) which encodes the depth information of the scatterers in Fourier domain OCT.

**Fig. 8.**
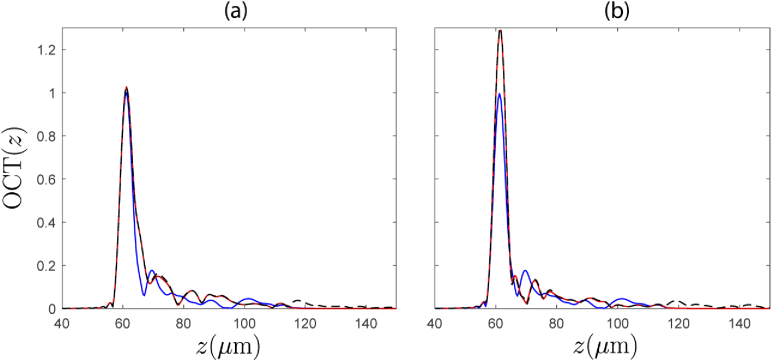
OCT A-scans generated from the isolated field of a point scatterer located at the position (x,y,z)=(0,0,61.75μm) generated with pseudo-plane wave illumination with tilt angle $\theta_{i}=0.0069$ radians (a). Tilt angle of $\theta_{i}=0.0762$ radians (b). Red lines show the directly evaluated A-scan, blue lines show the A-scan formed from the synthesized field. Black dashed lines show a "corrected" synthesized A-scan using knowledge of the field arriving at the point scatterer.

**Fig. 9.**
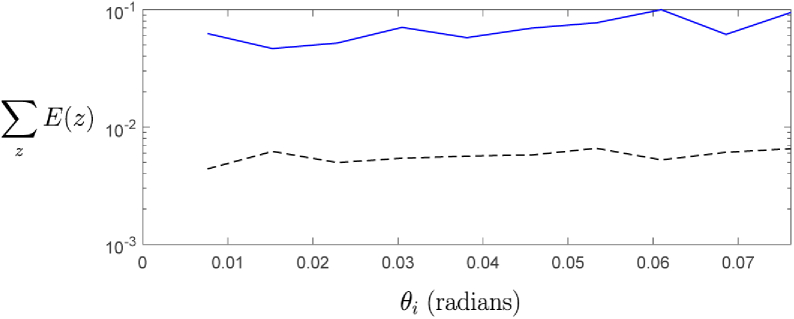
Error metric summed over all depths for the A-scans shown in Fig. 8. Each line shows the error as a function of the tilt angle of the illuminating wave. (Blue) - Synthesized field. (Black dotted) - “Corrected” synthesized field using directly evaluated information of the field incident on the point scatterer.

In addition to these analyses, we also investigated the propagation of the scattered field from the same z=61.75μm plane back towards the detector, and the dependence of this returning field on the above mentioned discrepancy between the directly evaluated and synthesized fields at the z=61.75μm layer. This was achieved by performing simulations with an additional point scatterer present at the z=61.75μm layer, and isolating the back-scattered field due to this point scatterer alone. Using this isolated field, an OCT A-scan was generated and compared between the directly evaluated signal, and the synthesized signal for different tilt angles of a weakly focussed beam. The A-scans were attained by computing the back-scattered field coupled into a single mode optical fiber, and calculating the interference with a reference signal. In Fig. 8(a) the generated A-scans are compared for a weakly focussed source beam with a low tilt angle of $\theta_{i}=0.0069$ radians. It is seen that the directly evaluated and synthesized fields (red and blue, respectively) show close agreement at the peak of the signal, yet show some disagreement beyond the peak. For the larger angle of incidence of the illuminating weakly focussed beam shown in Fig. 8(b), the disagreement increases, even at the peak of the signal. However, the location of the peak still remains centered on the point scatterer location at z=61.75μm. Also shown on the figures is a “corrected” synthesized A-scan, where a correction factor is applied uniformly over the isolated scattered field. This correction factor simply accounts for the directly evaluated incident field arriving at the point scatterer, rather than the assumed field that is used to generate the synthesized case. This corrected case (dotted black line) is seen to be much closer to the directly evaluated case for all regions except at the tail of the signal. As this correction requires knowledge of the exact field arriving in the plane of the scatterer, such a correction could not be applied in practice. However, what this does show is that if the field arriving at the scatterer is accurately accounted for, then the synthesized OCT signal is very close to the directly evaluated case. This suggests that the journey of the scattered field back through the sample towards the detector does little to further degrade the synthesized signal, and that it is primarily the discrepancy in the arriving field at the scatterer location that is the main downfall of the synthesized approach. In Fig. 9, we further compare the error metric evaluated for the synthesized A-scan of the isolated point scatterer, and the “corrected” synthesized A-scan. Again it is clear that the “corrected” synthesized field has significantly lower error (by an order of magnitude) than the synthesized field for all incident angles of the source, demonstrating that the most significant contributor to the error of the synthesized signal is the discrepancy in the field arriving at the scattering layer of interest, and not the journey of the scattered field back towards the detector.

**Fig. 10.**
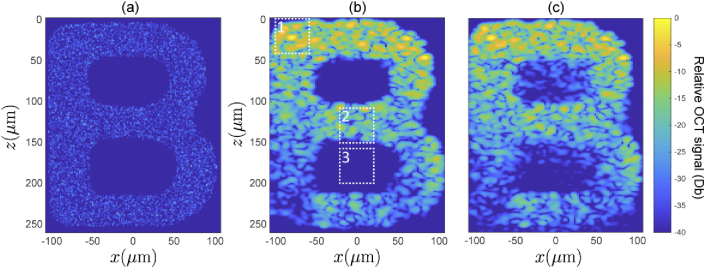
(a) Illustration of refractive index distribution, projected from entire extent of sample in the y-direction. (b) Reference and (c) synthesized OCT B-scans of the scattering object. Both images are normalized to the maximum value present in the scan, with the color bar showing the number of decibels the signal is below the maximum. Highlighted areas show regions which are displayed in more detail in Fig. 11.

**Fig. 11.**
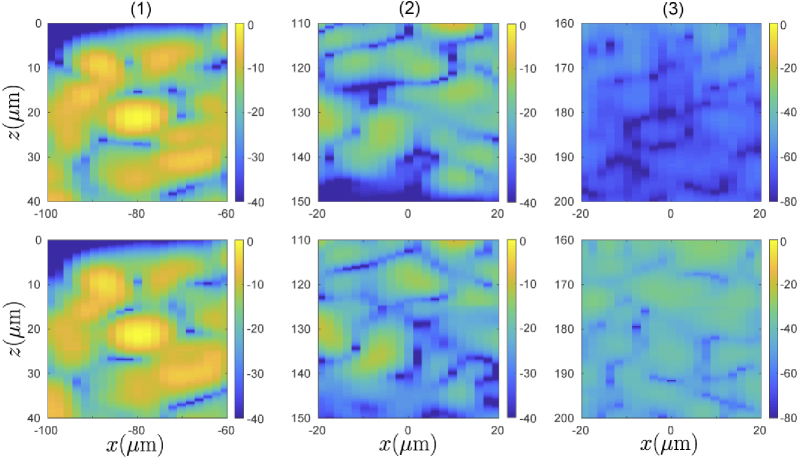
Close-up comparisons of reference OCT B-scan (top row) and synthesized OCT B-scan (bottom row) over the three regions outlined in Fig. 10(b). Region 1 (column 1). Region 2 (column 2). Region 3 (column 3).

Finally, we now evaluate the accuracy of a full synthesized OCT B-scan of the scattering object illustrated in Fig. 10(a). Figure 10(b) shows a reference B-scan simulated by direct evaluation of the scanning-mode PSTD-based image formation model. This reference image required 110 separate PSTD simulations; one for each of the lateral scan-positions with a spacing of 1.95μm between each. The source beam in this computation was identical to that used to generate the PSF in Fig. 2 (objective focal length $f_{2}=36$mm, aperture $R_{a}=3.5$mm). Full details of this simulation can be obtained from [38]. Figure 10(c) shows the B-scan obtained using the synthesized field method described in Section 2, where only one PSTD simulation was performed for a normally incident plane wave. The field synthesis algorithm was applied so as to build up the OCT B-scan for all lateral scan positions considered in the directly evaluated B-scan, and is composed of segments of images reconstructed for assumed scatterer depths of $z_{s}$ = 0,50,100,150,200 and 250 μm, in the same manner as in the PSF reconstruction above. Both Fig. 10(b) & (c) are plotted as $10\;{\text{log}}_{10}(|\text{OCT}{|}^{2})$, where the OCT signal is defined in Eq. (5), and are plotted over a 40 decibel range where the images are both normalized to their respective maximum value. The resulting images, including the speckle structure, are consistent at shallow depths. The level of agreement between the two degrades as imaging depth increases. We also see that the holes in the letter “B” have somewhat less well defined edges in the synthesized case compared with the directly evaluated case. The three highlighted regions in Fig. 10(b) & (c) are compared in finer detail in Fig. 11. In Region 1, near the upper surface of the image, the speckle structure appears to be closely replicated in the synthesized case. A faint line may be seen along the z=25μm line, where the image has been stitched together from two reconstructions using different values of $z_{s}$, otherwise it is difficult to distinguish the two methods. In Region 2 which is at a depth of approximately 130μm, The two methods begin to show some differences, while still maintaining a high degree of correlation between the two speckle patterns. Furthermore, the relative magnitudes of the signals are also still closely related. In Region 3, at a depth of approximately 180μm, where there are no scatterers present, we begin to see more significant discrepancies between the two methods. Note that this region is plotted over a greater intensity range so as to allow for visualisation of the low signal levels. Both simulations reveal non-zero signals due to multiple scattering. However, in the synthesized case the magnitude of the multiple scattering contributions are significantly higher. This is expected to be a result of the fact that in the synthesized case the sample is illuminated by a plane wave rather than a focussed beam, meaning that there are more paths that multiply scattered light can take and still fall within the synthesized aperture for a particular scanning location. If each plane wave making up the focussed beam were simulated directly using a PSTD simulation, the resulting B-scan would be identical to the directly evaluated (reference) case due to the linearity of Maxwell’s equations. However, as discussed in Sec. 3.3.1, an error is introduced into the phase of the fields synthesized for non-zeros angles of incidence when only a single PSTD simulation (for a normally incident plane wave) is used. This error in the phase means that an amplitude error results when the contribution due to each plane wave is summed. It is this error which leads to an increased multiply scattered component in the synthesized B-scan.

**Fig. 12.**
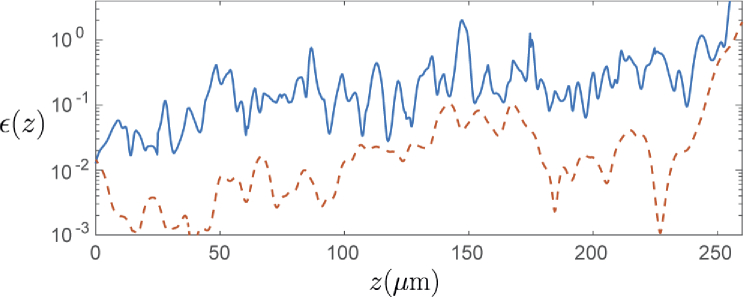
Error metric ϵ(z) taken over the width of the “spine” of the letter B in the x-direction, where there are scatterers present at all depths (up to 250 μm). No smoothing (solid blue). Smoothing over a 20×20μm window applied to each OCT image before computing the metric ϵ(z) (dotted red).

Figure 12 shows a plot of the error metric (Eq. (6)) evaluated over an 18 micron wide window in the x-direction (centered on x=−80μm), and over the entire depth of the sample in the z-direction. This region covers the spine of the letter “B” , and has a continuous presence of scatterers down the entire depth of the object. Here we see that the error (blue line) increases as a function of depth, beginning in the vicinity of $10^{-2}$ at the top surface of the sample, and growing to ≈0.7 by the lower section of the sample. Note that the error increased very sharply at the bottom of the image due to dividing by a small normalising quantity where the sample ends. Also on this graph we have plotted the error metric evaluated over the same region, but where the OCT images were each averaged over a 20×20μm wide sliding window prior to evaluation (dashed-red line). This error metric evaluated over the smoothed data shows how similar the two methods are in a situation where the speckle structure is not of importance, but rather the mean image magnitude. The error of this smoothed data stays below approximately 0.01 for the first 100 microns, and below 0.1 for the rest of the sample.

## 4. Discussion and conclusion

We have shown that OCT images can be approximately synthesized using only one PSTD simulation which employs normally incident plane wave illumination. Such images are usually simulated by evaluating one PSTD simulation for each scan position of the focussed beam. The motivation for investigating this approximate approach is a significant reduction in computational resources required by to the PSTD algorithm, which is the most computationally intensive part of the OCT image formation model. For instance, the directly evaluated OCT image in Fig. 10(b) required 110 PSTD simulations each with a width of 270 PSTD grid cells (i.e., Yee cells) in the x-direction. This gives an effective combined width of all simulations of ≈30000 grid cells, and required 2970 hours of computation time running 20 OpenMP threads on a Intel Xeon Gold 6148 CPU @ 2.40GHz. The single plane wave simulation from which Fig. 10(c) was synthesized had a lateral width of only 1422 grid cells in the x-direction (the actual width of the sample), and required only 147 hours on the same hardware. The post-processing required for the image synthesis required 7 minutes per A-scan running 20 threads in Matlab on the same hardware. The total post-processing time was 7 minutes × 110 A-scans × 6 values of $z_{s}$ = 77 hours. This gives a total computation time to produce Fig. 10(c) of 224 hours, versus 2970 hours for the reference in Fig. 10(b), and thus over a factor of 13 less computation time required using the proposed approach.

We have also demonstrated, however, that this reduction in computational requirements comes at the cost of two sources of error which lead to a loss of accuracy in the synthesized images. The first of these errors relates to how the forward propagating field within the sample is assumed to change as the angle of propagation of plane wave illumination changes. In particular, the phase of the forward propagating field within the sample is assumed to change identically to the free-space case as the angle of propagation of plane wave illumination is varied. The second source of error is the increased contribution from multiple-scattering in the synthesized image when compared to the directly evaluated image. Both of these sources of error grow as a function of depth within the sample. For sparsely populated or weakly scattering samples, the influence of these errors are likely to be sufficiently small to justify the gain in efficiency using the proposed field synthesis approach. However for denser samples, such as the one described in the previous section, the errors may become more significant, depending on the application. In particular where high sensitivity simulations are performed, the erroneous multiple scattering may not be acceptable. This approach may also not be suitable for applications where the correct speckle structure is important, since in deep regions of a densely scattering sample, the speckle structure of the synthesized image begins to deviate from that of the directly evaluated image. However, the field synthesis approach may be acceptable for applications where only speckle statistics and macroscopic structure are the most important simulation features.

Due to the significant computational burden of full-wave rigorous electromagnetic scattering models (such as PSTD or FDTD), exploration of such approaches to improve efficiency are an important direction of investigation, potentially enabling faster development of these models of OCT image formation. With increased efficiency also comes the ability to model larger samples which have greater relevance to biomedical imaging applications, and to explore larger sets of variables. An additional benefit of the method described here is that we can synthesize arbitrary source-detector configurations without needing to re-run the PSTD code, allowing for rapid investigation of various experimental configurations.
